# Commentary: Therapeutic Potential of Targeting the Auto-Inhibition of ASIC1a for Neuroprotection Against Ischemic Brain Injury

**DOI:** 10.3389/fphar.2020.604892

**Published:** 2020-11-24

**Authors:** Matthew William, Sejla Turnadzic, Xiang-Ping Chu

**Affiliations:** Department of Biomedical Sciences, School of Medicine, University of Missouri-Kansas City, Kansas City, MO, United States

**Keywords:** acid-sensing ion channel, acidosis, neuroprotection, auto-inhibition, cerebral ischemia

## Introduction

Strokes are the fifth leading cause of death and disability in the United States (Zhou et al., 2018). This hypoxic event forces surrounding tissue to switch to anaerobic glycolysis and produce lactic acid, leading to acidosis (Rehncrona, 1985). The primary ion channels responsible for sensing acidosis are acid-sensing ion channels (ASICs) (Waldmann et al., 1997). Studies have shown that ASICs are involved in both physiological and pathological conditions (Chu and Xiong, 2012; Kellenberger and Schild, 2015). Among ASICs, the ASIC1a subtype is most sensitive to acidosis; it has high expression in the brain, and activation reveals calcium permeability (Xiong et al., 2004; Chu et al., 2014; Gründer and Pusch, 2015). We have shown that activation of ASIC1a during brain ischemia triggers neuronal injury, and deletion of ASIC1a generates neuroprotection (Xiong et al., 2004). Activation of ASIC1a also induces membrane depolarization (Jiang et al., 2009), subsequently leading to activation of other ion channels and receptors such as the *N-methyl-D-aspartate* (NMDA) receptor. Thus, ASIC1a-NMDA receptor interaction contributes to acidosis-mediated ischemic brain injury (Gao et al., 2005;Isaev et al., 2008). Recently, studies from the Xu laboratory have demonstrated an additional mechanism by which acidosis could induce neuronal necroptosis through activation of ASIC1a, one independent of its ion-conducting function (Wang et al., 2015). They reported that acidosis caused receptor-interacting serine/threonine-protein kinase 1 (RIPK1) to interact with the C terminus (CT) “death motif” of ASIC1a protein, triggering phosphorylation of RIPK1 and resulting in neuronal necroptosis, independent of intracellular calcium levels. This demonstrated the crucial involvement of ASIC1a-RIPK1 activation in ischemic brain injury.

## ASIC1a Auto-Inhibition Prevents Neuronal Necroptosis

A recent study reported in the *Nature Communications* (Wang et al., 2020) from the Xu laboratory expanded their previous findings and found that the cytoplasmic N terminus (NT) and CT of the ASIC1a protein interact in order to prevent necroptosis under normal physiological conditions. They showed that when an exposed derivative protein of the CT (known as CP-1-2) was released into cortical neurons, it recruited RIPK1 and induced necroptosis, even at physiological pH. They hypothesized that the CT of ASIC1a binds to the NT at physiological pH, preventing it from leading to necroptosis, like CP-1-2 did. Using the Förster resonance energy transfer technique, they observed the interactions between the CT and the NT and found that exposure to an acidic solution caused the masked NT and CT to separate. Returning to physiological pH of 7.4 reversed this separation. From this observation, they speculated that acidosis causes the CT and NT to unbind which lead to the binding of CT to RIPK1, resulting in necroptosis. To prove this prediction, they truncated the NT off in ASIC1a and found that neurons with this truncation underwent necroptosis at physiological pH, further suggesting that the NT binds to the CT under non-acidic conditions, inhibiting necroptosis. Furthermore, they examined the free NT, which spontaneously binds to a protein known as N-ethylmaleimide-sensitive fusion ATPase (NSF) during acidosis. They found that when NSF binds to the free NT, it prevents it from rebinding to the exposed CT, thus allowing the free CT to interact with and activate RIPK1, triggering necroptosis. To test this hypothesis, they used shRNA to knockdown the NSF protein and found that the neurons with less NSF expression revealed an attenuated acidosis-induced necroptosis. They also examined four negatively charged glutamate residues, which are able to bind to positively charged lysine residues on the CT death motif during physiological conditions, on the distal NT of ASIC1a protein. By mutating the glutamate residues to alanine, they fully removed all electrostatic interaction between the NT and CT. Freeing of these two ends resulted in increased levels of necroptosis. In order to prevent necroptosis during acidosis, they synthesized the peptide NT_1-20_, mimicking the NT of ASIC1a and found that when CP-1-2 was introduced into neurons, NT_1-20_ was able to bind to it, preventing RIPK1 activation, and blocking necroptosis. Pretreatment with the NT_1-20_ peptide before exposing ASIC1a to pH 6.0 also significantly attenuated necroptosis. Finally, they injected the peptide NT_1-20_ into mouse lateral ventricles and found that it significantly reduced ischemic brain damage in an experimental stroke mouse model. In this studies, tags were added to the synthetic NT_1-20_ and CT ASIC peptides in order for them to get into to cytoplasm of the cells and have their effect. Collectively, their study suggests that synthetic peptides, such as NT_1-20_, may mimic auto-inhibition of ASIC1a, effectively attenuating acidosis-induced necroptosis during ischemic brain injury.

## Discussion

Although previous studies have examined how ASIC1a induces neuronal damage in stroke (Xiong et al., 2004; Gao et al., 2005; Zhou et al., 2019), multiple sclerosis (Vergo et al., 2011), and spinal cord injury (Mazzone et al., 2017), this study from the Xu laboratory opens a new avenue to a potential therapeutic pathway by targeting ASIC1a auto-inhibition (Wang et al., 2020). Future studies are needed to investigate agents that could target the ASIC1a auto-inhibition pathway. One potential subject is the use of NT_1-20_ in preventing neuronal death. While NT_1-20_ seems like a promising peptide in preventing the CT from recruiting RIPK1, other functions of the peptide should also be examined. The NSF protein plays a critical role in fusing synaptic vesicles (Whiteheart et al., 1994; Belluzzi et al., 2016), so it is important to explore the consequences of inactivating it. Because knockdown of the NSF protein significantly reduced acidosis-induced necroptosis, it could be a potential therapeutic target against ischemic brain injury, but the consequences of inhibiting the NSF protein should be further investigated as it may interfere with synaptic vesicle fusion. RIPK1 is known to play a critical role in ASIC1a-mediated necroptosis by binding to the CT during ASIC1a activation (Wang et al., 2015). Selective inhibitor of RIPK1 such as necrostain-1 has been applied in stroke (Zhang et al., 2016), traumatic spinal cord injury (Wang et al., 2014), and amyotrophic lateral sclerosis (Re et al., 2014; Ito et al., 2016) and reveals protection in CNS diseases (Degterev et al., 2019; Yuan et al., 2019). The efficacy and selectivity of both direct ASIC inhibitors and RIPK1 inhibitors in preventing ASIC1a from committing acidosis-induced necroptosis should be studied carefully, as such inhibitors have had promising results in other pathways mediated by RIPK1 (Mifflin et al., 2020). ASIC1a plays an important role on its ion-conducting function such as synaptic plasticity, learning, and memory (Huang et al., 2015). Studies from the Xu laboratory uncovered an ion conduction-independent function of ASIC1a responsible for ischemic brain injury. Their study sheds new lights on potential therapeutic intervention of ASIC1a-mediated ischemic brain injury. Thus, targeting the auto-inhibition of ASIC1a without affecting its physiological function becomes a desirable strategy in treatment of stroke patients.

## Author Contributions

All authors listed have made substantial, direct and intellectual contribution to the work, and approved it for publication.

## Funding

The work was supported by grant from American Heart Association (19AIREA34470007) to XC.

## Conflict of Interest

The authors declare that the research was conducted in the absence of any commercial or financial relationships that could be construed as a potential conflict of interest.
